# Prospective Associations between Religiousness/Spirituality and Depression and Mediating Effects of Forgiveness in a Nationally Representative Sample of United States Adults

**DOI:** 10.1155/2012/267820

**Published:** 2012-05-22

**Authors:** Loren L. Toussaint, Justin C. Marschall, David R. Williams

**Affiliations:** ^1^Department of Psychology, Luther College, 700 College Dr., Decorah, IA 52101, USA; ^2^Department of Society, Human Development, and Health, Harvard University, Boston, MA 02115, USA; ^3^Department of African and African American Studies, Harvard University, Cambridge, MA 02138, USA; ^4^Department of Sociology, Harvard University, Cambridge, MA 02138, USA

## Abstract

The present investigation examines the prospective associations of religiousness/spirituality with depression and the extent to which various dimensions of forgiveness act as mediating mechanisms of these associations. Data are from a nationally representative sample of United States adults who were first interviewed in 1998 and reinterviewed six months later. Measures of religiousness/spirituality, forgiveness, and various sociodemographics were collected. Depression was assessed using the Composite International Diagnostic Interview administered by trained interviewers. Results showed that religiousness/spirituality, forgiveness of oneself and others, and feeling forgiven by God were associated, both cross-sectionally and longitudinally, with depressive status. After controlling for initial depressive status, only forgiveness of oneself and others remained statistically significant predictors of depression. Path analyses revealed that religiousness/spirituality conveyed protective effects, prospectively, on depression by way of an indirect path through forgiveness of others but not forgiveness of oneself. Hence, forgiveness of others acts as a mechanism of the salutary effect of religiousness/spirituality, but forgiveness of oneself is an independent predictor. Conclusions regarding the continued development of this type of research and for the treatment of clients with depression are offered.

## 1. Introduction

The present study examines the associations of religiousness/spirituality with depression in a nationally representative sample of United States adults. A parallel aim is to investigate the extent to which various dimensions of forgiveness may act as mechanisms of the connection between religiousness/spirituality and depression. Though research on religiousness/spirituality and its connections to depression and broader mental health has proliferated for several decades, only a small fraction of this research has utilized longitudinal designs and population-based samples. The present study aims to address this void in the literature and contribute to our understanding in this area.

 In the *Handbook of Religion and Health*, Koenig et al. [1] define religion as “an organized system of beliefs, practices, rituals, and symbols” (page 18) intended to encourage a close relationship with God or higher power/truth/reality and to help individuals understand their connection to others living in a community. Spirituality is defined as the search for understanding and meaning in life that may or may not be related to religious rituals and community [1]. Koenig et al. devote an entire chapter in this handbook to the review of published research on religiousness/spirituality and depression. These authors provide an overall synopsis of this literature and suggest that generally religious and spiritual individuals experience less depression, as compared to non-religious/spiritual individuals. Over 100 studies are indexed that on the whole support this conclusion. The size of this correlation is modest, but not trivial, and meta-analytic reviews have estimated the overall effect size to be in the neighborhood of *r* ≈ .20 [2]. In about a decade since the publication of this comprehensive review, more than 60 studies have been added to this literature that again support the conclusion that religiousness/spirituality shows salutary associations with mental health [3]. As one example, recent longitudinal work showed that church attendance was associated with reduced development of subsequent depressed mood over the course of eight years in older Australian adults [4]. Despite sustained interest in the potentially beneficial effects of religiousness/spirituality on mental health, few studies have examined the question using similar longitudinal designs or population-based samples. Hence, there remains a gap in the literature that cannot be filled by cross-sectional analyses of convenience samples. Prospective designs are needed to discern the temporal ordering of the association between religiousness/spirituality and depression. Evaluating the generalizability of any such effect will require representative, population-based samples. The present study offers both of these advantages.

 In addition to testing the prospective relationship between religiousness/spirituality and depression, the present study offers the opportunity to examine several dimensions of forgiveness as potential mechanisms of this association. A number of psychosocial, health, and health-behavior mechanisms have been invoked to explain the salutary associations of religiousness/spirituality and depression; yet the question of what accounts for these effects has yet to be entirely answered [5]. Perhaps forgiveness is a viable mechanism.

Forgiveness is a multidimensional phenomenon that involves the voluntary letting go of negative thoughts, feelings, and behaviors and potentially even replacing these with positive thoughts, feelings, and behaviors [6]. Often the focus of forgiveness is toward another individual, but the focus of forgiveness can also involve oneself [7], God/higher power [8], and sometimes involves the process of seeking the forgiveness of others [9]. Forgiveness has been shown to be related to religious and spiritual variables, but often it is not related at the levels of magnitude that one might expect [10, 11]. Further, investigations of the connections between religiousness/spirituality and forgiveness almost exclusively regard forgiveness of others, and do not consider other dimensions of forgiveness. Nonetheless, there is a modest but reliable association between religiousness/spirituality and forgiveness of others and it may likely extend to other dimensions of forgiveness as well, especially feeling forgiven by God and seeking others' forgiveness.

 Given its association with religiousness/spirituality, forgiveness might be considered a viable mechanism of the salutary associations of religiousness/spirituality with depression if it too were associated with depression or other related mental health conditions. Indeed, associations between forgiveness and a variety of mental health outcomes have been documented [12]. A vast majority of these studies have examined cross-sectional associations in convenience samples or highly specific patient populations. A few exceptions do exist in which, for instance, forgiveness has been linked to diagnosable depression in a nationally representative sample of United States adults [13] or in a prospective fashion demonstrating that forgiveness predicts subsequent mental distress [14]. These recent findings suggest that forgiveness may have meaningful prospective associations with mental health outcomes.

 Given the research findings reviewed above, we believe that a prospective study of religiousness/spirituality, forgiveness, and depression will offer useful insights and add to what we estimate is only a handful of studies of this type. Based on our review, we have built a model to examine four hypotheses. First, we hypothesize that religiousness/spirituality will show a prospective, protective (inverse) association with depression. Second, we hypothesize that religiousness/spirituality will be positively associated with multiple dimensions of forgiveness. Third, we hypothesize that forgiveness will show a prospective, protective (inverse) association with depression. Fourth, we hypothesize that the protective (inverse) association of religiousness/spirituality with depression will operate via the mechanism of forgiveness. Put another way, we expect that religious and spiritual individuals will experience a greater proclivity toward multiple forms of forgiveness, and this tendency will in turn yield benefits for depression risk.

## 2. Method

### 2.1. Sample

 Participants responded to the Survey of Consumers, a telephone survey of adults age 18, and older conducted by the University of Michigan's Institute for Social Research. The sample was nationally representative and was randomly selected using the two-stage random-digit-dialing (RDD) procedure described by Waksberg [15]. The survey employs a rotating panel design to gather data from approximately 500 respondents on a monthly basis. Each monthly sample consists of about 300 new respondents and 200 respondents being re-interviewed six months after their initial interview. The initial sample for this study consisted of the new national sample selected each month for five months for a total of 1,423 respondents. The reinterview target sample consisted of approximately three-fourths of the original respondents who were randomly selected for a total of 1,055 respondents. Both the initial and reinterview samples are nationally representative. The response rate for the survey ranged from  .69 to  .71. After listwise deletion of missing data, the final sample consisted of 966 respondents who participated at both times 1 and 2.

### 2.2. Measures

#### 2.2.1. Depression

 The measurement of major depressive episode was based on the definitions and criteria specified in the revised edition of the American Psychiatric Association's Diagnostic and Statistical Manual of Mental Disorders (DSM-III-R). Major depressive episode was assessed using a brief, screening version [16, 17] of the depression module of the World Health Organization's (WHO) Composite International Diagnostic Interview, Version 1.0 (CIDI) [18, 19]. The CIDI is a structured interview schedule designed to be used by trained interviewers who are nonclinicians to assess the prevalence of specific psychiatric disorders [20]. WHO field trials and other methodological studies have shown good test-retest reliability and clinical validity of these CIDI diagnoses [21, 22].

#### 2.2.2. Forgiveness

 Four dimensions of forgiveness were assessed, and, as with all following scales, all scoring was done so that higher scores represented higher levels of the construct. All reported alpha values for forgiveness and all following scales are based on the present study's data. Forgiveness measures used in the present investigation were adapted from those originally used by Watson et al. [23, 24], Mauger et al. [25], Idler et al. [26], and Gorsuch and Hao [27]. For a complete list of the items utilized to measure forgiveness, see Table 1.

#### 2.2.3. Religiousness/Spirituality

4 religiousness/spirituality factors were assessed as follows: *service attendance* was measured by asking respondents how often they went to religious services. Response categories ranged from (1) never to (6) more than once a week. Frequency of *prayer* was measured by asking how often they prayed in places other than church and synagogue. Responses ranged from (1) never to (6) more than once a day. Respondents were also asked to rate how *religious* and how *spiritual* they were on a ten-point scale.

The four religiousness/spirituality items were combined to form a single index of religiousness/spirituality. This decision was made for five reasons. First, the items had virtually identical patterns and magnitudes of association with depression. Second, when factor analyzed (maximum likelihood extraction) the items loaded on a single latent factor (loadings = attend (.67), pray (.71), how religious (.92), and how spiritual (.80)). Third, using a single religiousness/spirituality composite offered parsimony in testing more complex religiousness/spirituality→forgiveness→depression mediation models. Fourth, multiitem composites contain less measurement error than single items. Finally, Koenig et al. caution researchers about multicollinearity in the use of multiple religiousness/spirituality indices in regression models. The average correlation between religious/spiritual variables in this study was *r* = .55, similar to the magnitude of the associations used to illustrate multi-collinearity problems in Koenig et al.'s [1] discussion. Using a composite instead of individual measures of religiousness/spirituality eliminates potential multi-collinearity issues in our regression models. The religiousness/spirituality composite index had an internal consistency of  .85.

#### 2.2.4. Control Variables

 Covariates assessed included: gender (male = reference category), age (in years), race (white = reference category), marital status (0 = not married; 1 = married), education (years completed), and income (13-point continuum ranged from under $10,000 to $100,000 or more). Exploratory analyses revealed that there were no noticeable differences between this coding scheme and other schemes that included dummy variables for separated and divorced, never married, and widowed respondents.

### 2.3. Statistical Analyses

 Data were weighted for age, gender, and race to take into account differential probabilities of selection and to adjust the demographics of the sample to that of the United States population using the Current Population Survey. Analyses proceeded in three phases. First, we computed and examined descriptive statistics (means/counts and standard deviations/ranges) and bivariate correlations. Second, we used a hierarchical logistic regression model to examine the prospective relations of religiousness/spirituality and forgiveness with depressive diagnosis six months later. This model was structured so that on step one depressive diagnosis at time one was entered. On step two, sociodemographic control variables were entered. On step three, the religiousness/spirituality index was entered. On step four, the four forgiveness variables were entered. In this way, religiousness/spirituality was tested for its longitudinal association with depressive status, and initial evaluations of the mediating effects of forgiveness were also considered.

 The third phase of analysis involved a more thorough and sophisticated examination of the mediating mechanisms that might explain the prospective relationship of religiousness/spirituality with six-month depressive diagnosis. Although the design of the hierarchical logistic regression model allows one to examine the extent to which the religiousness/spirituality coefficient is accounted for by forgiveness variables entered on the subsequent step, it does not allow for a specific test of the indirect effect of religiousness/spirituality through forgiveness to six-month depressive status.

Traditionally, a hierarchical model of this type might have been seen as sufficient for establishing that forgiveness acts as a mediating mechanism, but thanks to recent work [28], it has become clear that the traditional mediation approach is limited in two important ways. First, the traditional mediation approach requires a statistically significant total effect between religiousness and depressive diagnosis to be present. Second, it does not guarantee an explicit test of the indirect effect. A better manner in which to proceed is to simply test the indirect effects of interest [28]. However, when the outcome variable is dichotomous, calculating an indirect effect requires that any logistic regression coefficients in the estimated mediation model be standardized. There are at least six different methods for standardizing logistic regression coefficients, but an efficient and effective method requires simply that the predictors be standardized before entry into the logistic model [29]. Following this requirement, our mediation models are constructed so that coefficients representing the associations between religiousness/spirituality and forgiveness are *unstandardized* ordinary least squares regression coefficients. Coefficients representing associations between forgiveness variables and depressive diagnosis are *standardized* logistic regression coefficients. The indirect effect is computed by multiplying the unstandardized coefficient for religiousness/spirituality predicting forgiveness with the standardized coefficient for forgiveness predicting depressive diagnosis. The indirect effect can then be tested for statistical significance using the Sobel method [30].

## 3. Results

Table 2 gives the means and proportions, standard deviations and ranges, and bivariate associations for all study variables. Looking at columns one and two shows the associations between all predictors and depression both cross-sectionally and six months lagged. Lagged associations showed that the strongest predictor of depression at time two was prior depression at time one. Religiousness/spirituality showed a modest protective association, as did feeling forgiven by God. Protection against depression offered by forgiveness of oneself and others was noticeably larger. Bivariate associations for religiousness/spirituality and forgiveness variables with depression were similar for cross-sectional and lagged analyses.

Intercorrelations between religiousness/spirituality and forgiveness are contained in the lower right-hand portion of Table 2. Religiousness/spirituality was positively correlated at moderate levels with forgiveness of others and feeling forgiven by God. Religiousness/spirituality showed its highest positive correlation with seeking forgiveness and its lowest positive correlation with forgiveness of oneself. Forgiveness of others was moderately, positively correlated with forgiveness of oneself, feeling forgiven by God, and seeking forgiveness. Forgiveness of oneself was modestly, positively correlated with feeling forgiven by God and not correlated with seeking forgiveness. Feeling forgiven by God and seeking forgiveness were moderately, positively correlated.

Also evident in the table are small cross-sectional associations of sex and age with depression. Women showed slightly greater risk of depression at time one, and older participants showed slightly less risk. Age and income also showed a small protective, prospective association with depressive diagnosis. Hispanics were at increased risk, prospectively, of developing a depressive diagnosis.

Table 3 summarizes the results of the hierarchical logistic regression analysis. Unlike the bivariate associations reported above, the logistic model allows for an examination of the unique, prospective predictors of depressive diagnosis at time two, controlling the effects of initial depressive diagnosis. Model one examined the association of time one depression with time two depression. The odds ratio indicated that individuals with likely depressive diagnosis at time one showed over 12 times increased odds of depression at time 2. Regarding sociodemographic predictors of depression at time two, Hispanic respondents showed 3.6 times increased odds of depression, whereas higher-income respondents showed  .08 times reduced odds of depression. The most theoretically meaningful results of the logistic regression model were those showing effects for religiousness/spirituality and forgiveness. Religiousness/spirituality showed no influence on the odds of depression at time two, but forgiveness of oneself and others showed  .28 and  .32 reduced odds of depression, respectively.

**Figure 1:**
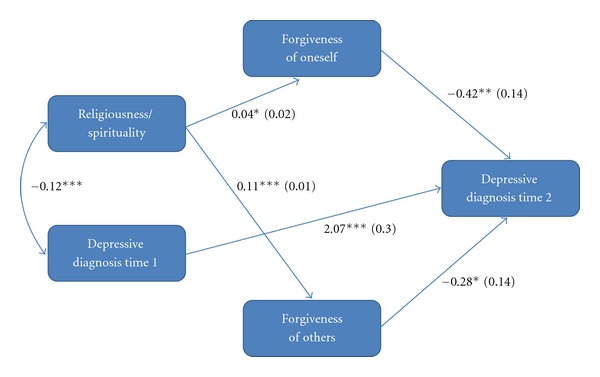
Path analysis of the prospective effects of religiousness/spirituality and prospective mediating effects of forgiveness on depression. Paths from religiousness/spirituality to forgiveness of oneself and others are unstandardized ordinary least-squares regression coefficients (standard errors). Paths from forgiveness of oneself and others to depression are standardized logistic regression coefficients (standard errors). All coefficients are net of the effects of gender, age, race, education, income, and marital status. *R* ^2^ = .32 for depression at time 2. Listwise *N* = 966.

 Based on the results of our bivariate and logistic analyses, we constructed a path model examining the indirect effects of religiousness/spirituality through forgiveness of oneself and others on depressive diagnosis at time two (see Figure 1). We did not include feeling forgiven by God or seeking forgiveness because neither variable had an independent direct effect on depression—a requirement for a mediating variable. The mediation model controlled the effects of depressive diagnosis at time one and all socio-demographic variables. The results of the model showed that religiousness/spirituality was significantly, positively associated with both forgiveness of oneself and others. Forgiveness of oneself and others were also significantly, negatively associated with depressive diagnosis at time two. The indirect effect of religiousness/spirituality through forgiveness of oneself on depressive diagnosis at time two was not statistically significant (*B* = .02, *Z* = −1.63, *P* = .10). The indirect effect of religiousness/spirituality through forgiveness of others on depressive diagnosis at time two was statistically significant (*B* = .03, *Z* = −1.98, *P* < .05). Hence, religiousness/spirituality shows a prospective, protective association with depression through forgiveness of others but not forgiveness of oneself.

## 4. Discussion

 This study set out to examine the prospective association of religiousness/spirituality with depression and the extent to which this association might be mediated through various dimensions of forgiveness. Our findings both confirm and disconfirm our expectations. As is typical in this type of design, the strongest predictor of depression at time 2 was depression at time 1. More importantly, bivariate analyses confirmed that religiousness/spirituality and forgiveness of oneself, forgiveness of others, and feeling forgiven by God were inversely associated with depression, and this was true both cross-sectionally and longitudinally. Unexpectedly, religiousness/spirituality did not show prospective associations with depression after controlling for initial depressive status in logistic models. As expected, forgiveness of oneself and others remained statistically significant, prospective predictors of depression, even after controlling for initial depressive status.

### 4.1. No Prospective Effect of Religiousness/Spirituality on Depression

Although unadjusted associations between religiousness/spirituality and depression do exist in the present study, religiousness/spirituality does not predict depression prospectively after adjusting for initial depressive status and other controls. The lack of a prospective association flies in the face of existing, sound empirical work suggesting the opposite [1–3, 5]. Furthermore, the results of the present study run contrary to those of dozens of studies showing that religiousness/spirituality offers protective advantages in multiple mental health domains [3]. As noted earlier, hundreds of other studies show salutary effects of religiousness/spirituality for physical health outcomes [1]. Given this outcome, there is good reason to consider what this might mean.

Although there are probably several reasons for the results observed in the present study, a couple of issues deserve discussion. First, as noted above, Koenig et al. [1] identified over 100 studies of religiousness/spirituality and depression that were conducted prior to 2001, and Toussaint et al. [3] identified an additional 66 that were conducted between 2001 and 2009. The majority of these studies reported at least one result suggestive of a protective connection between religiousness/spirituality and depression [3]. However, these studies have been overwhelmingly cross-sectional in nature, and most have relied on convenience samples. Koenig et al. [1] in their exhaustive review identified only 22 prospective cohort studies, and Toussaint et al. [3] identified little more than a handful of additional prospective studies in recent years. Of the 22 prospective cohort studies identified by Koenig et al. [1], only four utilized population-based samples, and in two of the four studies the beneficial effects of religiousness/spirituality were confined to a particular group (e.g., blacks, men). As such, the overreliance on cross-sectional methods to inform us about the relationships between religiousness/spirituality and depression seems a risky enterprise. Longitudinal research continues to be badly needed. The present study offers exactly that, a prospective examination of associations between religiousness/spirituality and depression in a representative sample of United States adults. Given how much faith we have placed in cross-sectional studies and how few and nuanced the prospective relations are, perhaps the current findings are not all that surprising. Rather, the present study might offer an initial point of reference for future prospective, population-based work.

A second issue in the present study regards the measurement of religiousness/spirituality. There has been great debate regarding what is the core essence of religiousness/spirituality and how it should be measured. Nevertheless, three key constructs have emerged [1, 31]. These include organizational religiousness, private religious practices, and religious importance. Indicators of each of these constructs were included in the present study; however, we chose to combine each of the items into an overall religiousness/spirituality index. Some may question the utility of this approach arguing that these are different constructs worthy of individual investigation. We would agree. However, as we indicated in the methods, there are several reasons for this decision. First, the direction and magnitude of the effects were all highly similar for each of the individual items. Second, all items factored onto a single factor that resulted in an index with good internal consistency. Third, examining single-item indicators has an untoward effect on predictive efficiency due to increased measurement error, and including multiple variables in regression models increases the likelihood of multicollinearity problems. Fourth, although we captured multiple dimensions of religiousness/spirituality, we used single-item indicators of each construct and did not comprehensively capture all relevant dimensions of religion/spirituality. As a result, the decision was made to combine the items into a single index. This allowed for a more sensitive test of the association between religiousness/spirituality and depression and allowed us to focus on the important mediating effects of forgiveness without requiring 8 to 16 paths from initial religiousness/spirituality variables to mediating forgiveness variables. The end result of creating a religiousness/spirituality composite was a sensitive yet parsimonious model of the prospective relations between religiousness/spirituality, forgiveness, and depression.

### 4.2. Indirect Effects of Religiousness/Spirituality on Depression Operate through Forgiveness of Others but Not Oneself

 The absence of a prospective relationship between religiousness/spirituality and depression offered the opportunity to refocus on an important and vexing question. That question is why might religiousness/spirituality have salutary effects? Though numerous psychological, social, and health mechanisms have been proposed and examined [1, 5, 32], the answer to why religiousness/spirituality has mental health benefits has not been entirely answered. Based on the present findings, we believe that forgiveness is an important piece of the puzzle.

 Our data show that religiousness/spirituality promotes forgiveness of others, which in turn has a moderate protective relationship with depression, and our path model bears out that this indirect effect is statistically significant. This is not so for forgiveness of oneself. In this case, the connection between religiousness/spirituality and forgiveness of oneself is not sufficiently large, even though it is statistically significant, to result in a significant indirect effect. It is clear that the indirect effect breaks down in this connection and not elsewhere, because the connection between forgiveness of self and depression is almost twice the size of the connection between forgiveness of others and depression. Furthermore, the prospective direct effect of forgiveness of oneself on depression is over twice the size of the typical effect of religiousness/spirituality on depression (*r* ≈ .20) [2]. Clearly, there is a meaningful benefit of forgiveness of oneself in terms of depression. But interestingly, this type of forgiveness is not strongly driven by religiousness/spirituality.

 To summarize, the results of our path model suggest that religious and spiritual persons secure mental health benefits through their increased likelihood to forgive others but not because they forgive themselves. This may reflect the emphasis on forgiveness of others that is present in organized religion [11, 33, 34] and the relative lack of religious teaching on forgiveness of oneself [35]. It appears there is a stark delineation between the forgiving mechanisms that convey benefit of religiousness/spirituality to depression. That said, forgiveness of oneself remains the most powerful prospective predictor of depression, outside of preexisting depression. Clearly, this dimension of forgiveness deserves more attention.

 Forgiving oneself has clearly been “The Stepchild of Forgiveness Research” [7]. That said, recent conceptual and empirical work has outlined some of the key correlates/predictors of self-forgiveness and its likely outcomes. Hall and Fincham [7] provide what might be considered the most comprehensive review of self-forgiveness and discuss several constructs which are likely to be causal antecedents of self-forgiveness. These include causal attributions, offense severity, shame/guilt, empathy, perceived forgiveness from victim/higher power, and conciliatory behavior. Nowhere is there mention of religiousness/spirituality in this conceptual model. This is a striking irony, because the general notion of forgiveness is infused with such religious and spiritual overtones that for years its study was thought not to be appropriate within science [11]. Moreover, Barry [35] indicates that the Bible offers no instance in which self-forgiveness is discussed. Given this context, perhaps it is not surprising that forgiveness of oneself emerged as an independent prospective predictor of depression and did not act as a mediating mechanism of the influence of religiousness/spirituality.

Recent empirical work confirms the importance of self-forgiveness for mental health. Macaskill [36] examined self-forgiveness in two studies involving over 500 participants. In her path analyses, self-forgiveness consistently showed robust associations with mental health. Importantly, in one of her path models where both self- and other forgiveness were modeled simultaneously, self-forgiveness showed statistically significant and moderate associations with both mental illness symptoms and life dissatisfaction while other forgiveness did not show significant associations with either variable. While these were cross-sectional studies and causal effects cannot be inferred, they do provide confirming evidence of the particular importance of self-forgiveness in our path models predicting depression.

### 4.3. Limitations

 As with studies of this type, there are some limitations to this work. First, the number of religious/spiritual and forgiveness items could have been greater. This would have allowed for broader assessment coverage of these constructs and would have helped to reduce measurement error. Nonetheless, even after a decade of enthusiastic forgiveness research, we are hard pressed to find measures that provide equally efficient and broad coverage of these four different forgiveness dimensions. Second, the elapsed time span between initial data collection and followup was six months. Though this provides ample time for changes to occur in mental health status, diagnosable depression likely changes less in this period of time. A longer follow-up would allow for even greater change, more variability, and potentially more predictive power. Third, given the paucity of longitudinal studies utilizing population-based samples, we feel that the findings from the present study provide a useful contribution to the existing literature. Nevertheless, we would encourage future investigators to consider collecting data from three or more waves so that growth modeling and latent trajectories could be established. Our two time-point data are useful in establishing prospective effects but do not allow for this type of more sophisticated understanding of change, variability in change, and latent trajectory analysis. Fourth, religiousness/spirituality and forgiveness are both measured at time one, and as such it is not possible to infer causal direction. Though religiousness/spirituality are often thought to be causally antecedent to forgiveness [11], it is also possible that being a more forgiving person might influence one's tendencies toward a religious/spiritual life. Future work utilizing three panels of data collection would be advisable and would improve on mediation models of the present type. Fifth, it is important to consider that respondents reporting on religiousness, spirituality, and forgiveness can be influenced by social desirability. Finally, in looking at depression as the main outcome in this study, it would have been useful to have controlled for other co-morbid disorders.

## 5. Conclusions

 The present study provides a prospective, population-based analysis of relationships between religiousness/spirituality and depression. To our knowledge, approximately five other studies have investigated this question using prospective designs and representative, population-based samples. As a result of the scantiness of this literature, our understanding of the influence of religiousness/spirituality on depression is limited. Continued development of this literature will inform us about the extent to which religiousness/spirituality can truly be considered a causal factor that impacts the risk of depression or whether it is merely a side effect of psychiatric disturbance.

 Prospective relationships between multiple dimensions of forgiveness and depression were tested in our analyses. Forgiveness of oneself and others proved to be important predictors of depression. Forgiveness of self is an independent predictor not connected to religiousness/spirituality as a mediating mechanism. Forgiveness of others, however, was found to convey the beneficial effects of religiousness/spirituality to depression. This is most likely the first demonstration, of which we are aware, of the prospective relations between forgiveness of oneself and others and diagnosable depression risk.

 Continued attention to the connections between religiousness/spirituality, forgiveness, and depression will undoubtedly shed light on the causal linkages between these constructs. To the extent that religious and spiritual persons reap benefit from forgiveness of others but not of oneself, there may be potential for tailored patient-centered forgiveness therapy; that is, religious and spiritual clients may be helped more by addressing issues regarding forgiveness of others, whereas self-forgiveness may be more important for less religious/spiritual clients or nonreligious. Future work might do well to examine the interaction effects of religiousness/spirituality and forgiveness. If synergistic effects were observed, this might suggest the importance of client-centered forgiveness interventions. With continued attention, the importance of religiousness/spirituality and forgiveness for depression care and treatment will be better understood, and we will gain improved resolution on the implications of religiousness/spirituality and forgiveness in the treatment of depression and related mental illnesses.

## Figures and Tables

**Table 1:** Forgiveness scales, items, and alphas.

Scale (alpha)	
Forgiveness of self (*α* = .67)	
I often feel that no matter what I do now I will never make up	
for the mistakes I have made in the past.^a^	
I find it hard to forgive myself for some of the things I have	
done wrong.^a^	
Forgiven by God (*α* = .64)	
Knowing that I am forgiven for my sins gives me the strength to	
face my faults and be a better person.^a^	
I know that God forgives me.^a^	
Forgiveness of others (*α* = .72)	
When someone hurts you, how often do you hold resentment	
or keep it inside?^b^	
When someone hurts you, how often do you try to get even in	
some way?^b^	
When someone hurts you, how often do you try to forgive the	
other person?^b^	
I have grudges that I have held on for months or years.^a^	
I have forgiven those who have hurt me.^a^	
Seeking forgiveness (*α* = .64)	
How often do you ask God's forgiveness when the respondent	
had hurt someone?^b^	
How often do you ask the other person's forgiveness when the	
respondent had hurt someone?^b^	
How often do you pray for someone who had hurt the	
respondent?^b^	

^a^Response scale: strongly agree, agree, disagree, or strongly disagree.

^b^Response scale: never, hardly ever, not too often, fairly often, and very often.

**Table:** (a)

	Dep T2		Dep T1		Fem		Age		Blk		Hsp		Ed		Inc	
Depression time 1	.41	***	1.00													
Sex (female = 1)	.05		.09	**	1.00											
Age	−.08	*	−.10	**	.04		1.00									
Black (other = 0)	.02		−.03		−.03		−.10	**	1.00							
Hispanic (other = 0)	.10	**	.00		.09	**	−.14	***	−.07		1.00					
Years of education	−.03		−.01		−.06		−.11	**	−.09	**	−.07	*	1.00			
Income	−.12	***	−.07		−.16	***	−.11	**	−.14	***	−.10	**	.46	***	1.00	
Separated/divorced (other = 0)	.04		.02		−.05		.06		.05		.01		−.09	**	−.14	***
Widowed (other = 0)	−.01		−.04		.16	***	.37	***	.01		−.05		−.22	***	−.28	***
Never married (other = 0)	.05		.01		−.07	*	−.41	***	.21	***	−.02		.03		−.12	***
Religiousness/spirituality	−.09	*	−.12	***	.14	***	.17	***	.01		−.05		.01		−.04	
Forgiven oneself	−.25	***	−.28	***	.05		.00		−.12	***	.02		.22	***	.23	***
Forgiven others	−.25	***	−.30	***	.10	**	.13	***	−.08	*	−.04		.04		.03	
Forgiven by God	−.09	**	−.08	*	.14	***	.07	*	.06		−.02		−.11	**	−.06	
Seek forgiveness	−.04		−.04		.27	***	−.02		.15	***	−.02		−.09	**	−.16	***

Mean/proportion	.11		.12		.55		45.90		.10		.06		14.03		8.02	
SD/range	0-1		0-1		0-1		16.11		0-1		0-1		2.49		3.60	

**Table:** (b)

	Sep/Div		Wid		Nvr Mar		Rel/Sp		FS		FO		FG		SF
Separated/divorced (other = 0)	1.00														
Widowed (other = 0)	−.11	**	1.00												
Never married (other = 0)	−.20	***	−.14	***	1.00										
Religiousness/spirituality	−.01		.09	*	−.14	***	1.00								
Forgiven oneself	−.07	*	−.07	*	−.08	*	.07	*	1.00						
Forgiven others	−.02		.08	*	−.13	***	.32	***	.39	***	1.00				
Forgiven by God	.06		.07	*	−.14	***	.39	***	.10	**	.26	***	1.00		
Seek forgiveness	.02		.02		−.03		.57	***	.02		.32	***	.38	***	1.00

Mean/proportion	.15		.07		.19		5.28		4.03		4.03		4.70		3.87
SD/range	0-1		0-1		0-1		1.76		1.15		.74		.66		.99

*P < .05, **P < .01, ***P < .001. Listwise *N* = 966. Dep = depression, Fem = female, Blk = black, Hsp = Hispanic, Ed = years of education, Inc = household income, Sep/Div = separated/divorced, Wid = widowed, Nvr Mar = never married, FS = forgiven self, FO = forgiven others, FG = forgiven by God, and SF = seek forgiveness. Values in the “mean/proportion” row represent means for continuous variables and proportions for dichotomous variables; likewise, values in the “SD/Range” row represent standard deviations for continuous variables and range of possible values for dichotomous variables.

**Table 3:** Unstandardized logistic regression coefficients, standard errors, and odds ratios for the association of time 1 depression, sociodemographics, religiousness/spirituality, and Forgiveness Predicting Time 2 Depression.

	Model 1				Model 2				Model 3				Model 4			
	B	S.E.	OR		B	S.E.	OR		B	S.E.	OR		B	S.E.	OR	
Depression time 1																
Depressed	2.52	.26	12.46	***	2.54	.28	12.70	***	2.50	.28	12.21	***	2.07	.30	7.91	***
Not depressed (referent)	—	—	—		—	—	—		—	—	—		—	—	—	
Sex																
Female					−.01	.28	.99		.02	.28	1.02		.22	.30	1.24	
Male (referent)					—	—	—		—	—	—		—	—	—	
Age					−.01	.01	.99		−.01	.01	.99		−.01	.01	.99	
Race ethnicity																
Black					.25	.41	1.28		.26	.42	1.29		.17	.42	1.19	
Hispanic					1.28	.54	3.61	*	1.26	.54	3.54	*	1.36	.55	3.90	**
White (referent)					—	—	—		—	—	—		—	—	—	
Years of education					.05	.06	1.05		.05	.06	1.05		.09	.07	1.10	
Income					−.09	.04	.92	*	−.09	.04	.92	*	−.06	.05	.94	
Marital status																
Separated/divorced					.39	.37	1.48		.37	.37	1.45		.39	.39	1.48	
Widowed					.28	.59	1.32		.28	.59	1.32		.35	.62	1.42	
Married (referent)					—	—	—		—	—	—		—	—	—	
Never married					.24	.37	1.27		.18	.38	1.20		.16	.39	1.17	
Religiousness/spirituality									−.06	.08	.94		−.01	.10	.99	
Forgiveness of oneself													−.32	.12	.72	**
Forgiveness of others													−.39	.20	.68	*
Forgiveness by God													−.21	.20	.81	
Seeking forgiveness													.09	.20	1.09	

*P < .05, **P < .01, ***P < .001; listwise *N* = 966; model 1 pseudo *R* ^2^ = .22, model 2 pseudo *R* ^2^ = .26, model 3 pseudo *R* ^2^ = .26, model 4 pseudo *R* ^2^ = .31.
